# Immunogenicity and safety of concomitant and sequential administration of yellow fever YF-17D vaccine and tetravalent dengue vaccine candidate TAK-003: A phase 3 randomized, controlled study

**DOI:** 10.1371/journal.pntd.0011124

**Published:** 2023-03-08

**Authors:** Vianney Tricou, Brandon Essink, John E. Ervin, Mark Turner, Ian Escudero, Martina Rauscher, Manja Brose, Inge Lefevre, Astrid Borkowski, Derek Wallace

**Affiliations:** 1 Takeda Pharmaceuticals International AG, Zurich, Switzerland; 2 Meridian Clinical Research, Omaha, Nebraska, United States of America; 3 Center for Pharmaceutical Research Inc, Kansas City, Missouri, United States of America; 4 Advanced Clinical Research, Boise, Idaho, United States of America; 5 Takeda Vaccines Pte. Ltd., Singapore; 6 Takeda Vaccines Inc., Boston, Massachusetts, United States of America; National University Singapore Saw Swee Hock School of Public Health, SINGAPORE

## Abstract

**Background:**

Yellow fever (YF) vaccination is often mandatory for travelers to YF-endemic areas. The areas with risk of YF partially overlap with those of dengue, for which there is currently no recommended vaccine available for dengue-naïve individuals. This phase 3 study assessed the immunogenicity and safety of concomitant and sequential administration of YF (YF-17D) and tetravalent dengue (TAK-003) vaccines in healthy adults aged 18–60 years living in areas of the US non-endemic for either virus.

**Methods:**

Participants were randomized 1:1:1 to receive the following vaccinations at Months 0, 3, and 6, respectively: YF-17D+placebo, TAK-003, and TAK-003 (Group 1); TAK-003+placebo, TAK-003, and YF-17D (Group 2); or YF-17D+TAK-003, TAK-003, and placebo (Group 3). The primary objective was to demonstrate non-inferiority (upper bound of 95% confidence interval [UB95%CI] of difference <5%) of YF seroprotection rate one month following concomitant administration of YF-17D and TAK-003 (Group 3) compared with YF-17D plus placebo (Group 1). The secondary objectives included demonstration of non-inferiority of YF and dengue geometric mean titers (GMTs) (UB95%CI for GMT ratio <2.0), and safety.

**Results:**

900 adults were randomized. YF seroprotection rates one month post-YF-17D (Month 1) were 99.5% and 99.1% in Group 1 and 3, respectively, and non-inferiority was demonstrated (UB95%CI = 2.69% i.e. <5%). Non-inferiority was also demonstrated for GMTs against YF one month post-YF-17D, and against DENV-2, -3, and -4 (UB95%CI <2), but not DENV-1 (UB95%CI: 2.22), one month post-second TAK-003 vaccination. Adverse event rates following TAK-003 were consistent with previous results, and no important safety risks were identified.

**Conclusions:**

In this study, YF-17D vaccine and TAK-003 were immunogenic and well tolerated when sequentially or concomitantly administered. The non-inferiority of immune responses to YF-17D and TAK-003 was demonstrated for concomitant administration of the 2 vaccines compared to separate vaccination, except against DENV-1 but with GMTs similar to those observed in other TAK-003 trials.

**Trial registration:**

ClinicalTrials.gov identified: NCT03342898.

## Author summary

Dengue and yellow fever are diseases endemic to tropical regions, often occurring in the same areas. There are highly effective vaccines against yellow fever, but there remains an unmet need for a dengue vaccine which can be used in people regardless of previous dengue infection. As multiple vaccines are often given together, particularly in travel medicine, we investigated the immunogenicity and safety of co-administration and sequential administration of a yellow fever vaccine and a tetravalent dengue vaccine (TAK-003) in adults living in areas of the US non-endemic for either disease. Most participants did not have pre-existing antibodies to either disease. We found that there was no reduction in immune response to the yellow fever vaccine when given at the same time as the dengue vaccine. There was also no reduction in response to dengue serotypes 2, 3, and 4, but this could not be established for the response to serotype 1. When sequentially administered, temporary increases were observed in yellow fever seroprotection rates after receipt of TAK-003, and in dengue seropositivity rates after receipt of the yellow fever vaccine, but these differences among groups were no longer seen by the end of the study. Both vaccines were well tolerated with acceptable safety profiles.

## Introduction

Dengue, a disease caused by the dengue flavivirus (DENV), is endemic in 129 countries worldwide, and was named by the World Health Organization (WHO) as one of the top ten threats to human health in 2019 [1]. DENV is predominantly transmitted by *Aedes aegypti* mosquitoes, with four virus serotypes (DENV-1, -2, -3, and -4) broadly co-circulating in endemic regions [2]. While the distribution of *A*. *aegypti* has been increasing with global temperature changes, risk of DENV transmission remains predominantly in tropical regions of South-East Asia, Central and South America, the Caribbean, and Sub-Saharan Africa [3]. DENV causes a range of symptoms from mild flu-like disease to potentially fatal severe dengue in a small proportion of individuals. Annually, there are an estimated 58–96 million symptomatic DENV infections, with 10.5 million cases requiring hospitalization [2, 4].

Yellow fever (YF) virus, the archetypal flavivirus, is closely related to DENV and is predominantly transmitted by *Aedes* and *Haemogogus* mosquitoes [5]. YF is endemic to tropical regions of Sub-Saharan Africa and South America, leading to partial overlap with DENV distribution [6]. YF virus causes mild symptoms in the majority of patients, however, a small proportion may go on to develop severe hemorrhagic symptoms, with a high mortality rate of approximately 50% [5, 7]. Vaccines against YF have been used since the 1930s and currently the only type of YF vaccine available is based on the attenuated 17D strain, derived from a wild-type YF virus isolated in Ghana in 1927 [8]. The highly efficacious YF-17D vaccines are administered as a single subcutaneous (SC) injection and seroconversion rates of >90% are reported by most studies [9]. YF neutralizing antibodies have been shown to persist for at least 40 years following single vaccination, indicating lifelong protection for vaccine recipients [10]. In addition to providing individual protection to those potentially exposed to YF virus, YF vaccination prevents the international spread of the disease by reducing the risk of importing the virus to non-endemic areas that contain competent YF vectors. In line with International Health Regulations, many countries require proof of YF vaccination in travelers arriving from areas at risk of YF virus transmission [11].

While a highly effective vaccine against YF has been available for decades, there remains an unmet need for an effective vaccine against dengue which is suitable for use in individuals without prior exposure to the virus. TAK-003 is a tetravalent dengue vaccine candidate based on a DENV-2 backbone, which was originally designed and constructed by scientists at the Division of Vector-Borne Diseases of the US Centers for Disease Control and Prevention (CDC) [12]. The DENV-2 strain in the vaccine (TDV-2) is based on an attenuated laboratory-derived virus, DENV-2 PDK-53 [13]. Recombinant serotype 1, 3, and 4 viruses (TDV-1, -3, and -4) were created by substituting pre-membrane and envelope genes of the DENV-2 backbone with those of the corresponding serotype [12, 14, 15]. TAK-003 has been shown to be immunogenic, efficacious, and well tolerated in phase 1, 2, and 3 studies, irrespective of baseline serostatus [16–23]. Overall vaccine efficacy in preventing virologically confirmed dengue (the primary endpoint) was estimated as 80.2% (95% confidence interval [CI]: 73.3–85.3%) in the first 12 months of the ongoing phase 3 efficacy study in children and adolescents living in endemic areas of Asia and Latin America, with efficacies of 76.1% (68.5–81.9%) and 66.2% (49.1–77.5%) in baseline seropositive and seronegative participants, respectively, after the 18-month period defined for secondary efficacy endpoints [23, 24]. More recently, two-year and three-year follow-up data supported the long-term safety and efficacy of TAK-003 against dengue disease and hospitalization [25, 26].

The overlapping geographical distributions of YF and dengue mean that travelers to endemic areas could be offered both vaccines. Concomitant administration increases the likelihood that recipients will receive all the necessary vaccines [27]. However, the potential for interactions between YF-17D and TAK-003, together with the effect of serological cross-reactions from sequential flavivirus exposure needs to be assessed. Flavivirus cross-reactive antibodies have been observed in sera from patients hospitalized with severe dengue or Japanese encephalitis (JE), with ≥4-fold increases in antibody titers against dengue, JE, and/or YF viruses observed when convalescent sera were compared with acute phase [28]. Cross-reactions have also been observed in previous dengue vaccine studies. For example, immune interference has been observed in adult and pediatric trials with YF-17D and CYD-TDV, the only currently commercially available dengue vaccine, which is licensed for individuals with evidence of previous DENV infection, with concomitant administration leading to reduced geometric mean titers (GMTs) against DENV [29, 30]. In a previous phase 1 study of a monovalent DENV-2 vaccine, participants with previous YF exposure developed cross-reactive antibodies to all four DENV serotypes, compared with low seroconversion rates to DENV-1, -3, and -4 in YF-naïve participants [31]. Similarly, YF priming resulted in a broader antibody response in a separate study of monovalent DENV-1, -2, -3, and -4 vaccines [32]. However, pre-existing YF antibodies have also been shown to impair response to vaccination against tick-borne encephalitis [33], and concomitant administration of YF and measles, mumps, and rubella (MMR) vaccine led to reduced immunogenicity of both vaccines [34].

The aim of this study was to assess the immunogenicity and safety of TAK-003 and YF-17D administered either concomitantly or sequentially in healthy adults aged 18–60 years living in areas non-endemic for either disease.

## Methods

### Ethics statement

The study was performed in accordance with the International Council for Harmonization of Technical Requirements for Registration of Pharmaceuticals for Human Use Harmonized Tripartite Guideline and Good Clinical Practice (ICH E6(R2)-GCP) ethical principles that have their origin in the Declaration of Helsinki, and applicable regulatory requirements. The protocol, amendments, informed consent forms, and translations were approved by Schulman central institutional review board (Cincinnati, Ohio, USA) prior to commencement of the study. Written informed consent was obtained from all participants prior to enrollment. The study protocol and the statistical analysis plan are available online at ClinicalTrials.gov where the study was registered (NCT03342898).

### Participants

Healthy adults aged 18–60 years were eligible for enrollment in the study. Exclusion criteria included fever (≥38°C) within three days of vaccination; known hypersensitivity or allergy to the vaccine or components; contraindications to YF-17D vaccine receipt; any serious chronic or progressive disease; altered immune function; body mass index ≥ 35kg/m^2^; pregnancy or breastfeeding; participation in another clinical trial within 30 days; receipt of vaccines prior to enrollment (14 days for inactivated, 28 days for live vaccines); previous vaccination against any flavivirus or tick-borne encephalitis; current or previous flavivirus infection and ≥1 year living in a dengue-endemic area.

### Study design

**Fig 1:**
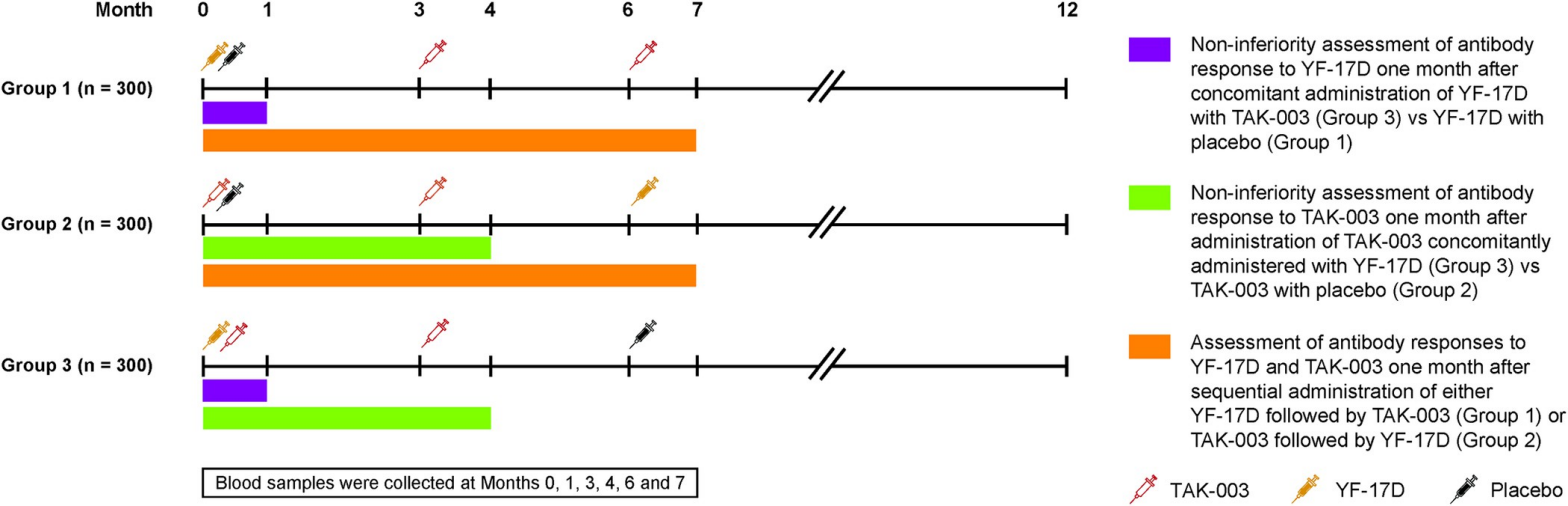
Study design. TAK-003, tetravalent dengue vaccine candidate; YF-17D, live attenuated yellow fever vaccine.

This phase 3, observer-blind, placebo-controlled randomized study was conducted at 11 sites across the US between February 2018 and May 2019. Participants were randomized 1:1:1 at enrollment using sequence of random numbers centrally-generated in blocks of 6 participants. The vaccine scheme for each of the groups is shown in **Fig 1**. Group 1 received YF-17D plus placebo at Month 0, TAK-003 at Month 3, and TAK-003 at Month 6. Group 2 received TAK-003 plus placebo at Month 0, TAK-003 at Month 3, and YF-17D at Month 6. Group 3 received TAK-003 and YF-17D at Month 0, TAK-003 at Month 3, and placebo at Month 6. This study design allowed for YF-17D and TAK-003 control groups up until the second injection at Month 3. Participants were followed for approximately six months following the third vaccination, leading to a total study duration of 12 months. All study participants, the site personnel responsible for the evaluation of the study endpoint, and relevant sponsor staff were kept blinded to the random assignment until completion of the study and database lock. Due to differences in the appearance of the study vaccines, vaccines were prepared and administered by qualified unblinded personnel who did not participate in any of the study assessments.

### Procedures

Blood samples were collected pre-vaccination at baseline (Month 0) and post-vaccination on Months 1, 3, 4, 6, and 7. Blood samples obtained at Months 3 and 6 were collected prior to the scheduled Month 3 and 6 vaccinations. Assessment of GMTs of YF neutralizing antibodies was performed using a validated plaque reduction neutralization test (Q2 Solutions, California, USA) at baseline and Months 1, 6, and 7, with GMTs corresponding to the dilution which resulted in a 50% plaque reduction (PRNT_50_). Seroprotection against YF was defined as reciprocal anti-YF neutralizing antibody titers ≥10 [35]. DENV neutralizing antibodies were assessed by microneutralization assay at all time points, with GMTs corresponding to the dilution which resulted in a 50% plaque reduction (MNT_50_) [14]. Participants were considered DENV seropositive if they had a reciprocal neutralizing antibody titer ≥10 against any of the four DENV serotypes. Participants were provided with diary cards to record solicited local and systemic adverse events (AEs) for 7 and 14 days after each vaccination, respectively. Unsolicited AEs were collected for 28 days following each vaccination, and serious AEs (SAEs) and medically attended AEs (MAAEs) were recorded throughout the study. AEs were assessed by the investigator for severity (mild, moderate, or severe) and relatedness to the study vaccination.

### Vaccines

TAK-003 was provided as a lyophilized formulation which was reconstituted with NaCl solution prior to administration. A single 0.5 mL dose of TAK-003 contained approximately 5.1, 4.5, 5.4, and 5.9 log_10_ plaque-forming units of TDV-1, TDV-2, TDV-3, and TDV-4, respectively. YF-17D (Stamaril) was also lyophilized and reconstituted with NaCl prior to injection. A single dose contained ≥4.74 log_10_ pfu YF-17D virus. While the licensed YF-17D vaccine in the US is YF-VAX (Sanofi Pasteur Inc, USA), Stamaril (Sanofi Pasteur Europe, France) was used in this study, in agreement with the FDA, as there was a shortage of YF-VAX at the time of the study. Normal saline for injection (0.9% NaCl) was used as placebo. Concomitant vaccines were administered into opposite arms.

### Objectives

The primary study objective was non-inferiority (NI) of the YF seroprotection rate one month after concomitant administration of YF-17D with TAK-003 (Group 3) vs YF-17D with placebo (Group 1).

Secondary objectives included assessments of NI of GMTs of neutralizing antibodies against individual DENV serotypes one month after the second dose of TAK-003 (Month 4) concomitantly administered with YF-17D vs TAK-003 with placebo (Group 3 vs Group 2) and NI of GMTs of neutralizing antibodies against YF, one month following concomitant administration of YF-17D with TAK-003 compared to YF-17D with placebo (Month 1; Group 3 vs Group 1).

Additional secondary immunogenicity objectives were seropositivity rates for individual DENV serotypes, one month post-second dose of TAK-003; GMTs of neutralizing antibodies and the seropositivity rates for all four DENV serotypes, one month post-first dose of TAK-003; GMTs of YF neutralizing antibodies and seroprotection rate in Group 2 one month post-YF-17D; GMTs of neutralizing antibodies and seropositivity rates for all four DENV serotypes one month post-second dose of TAK-003 in Group 1. Safety profiles of each vaccine were also assessed.

### Statistical analysis

The sample size of 300 participants per group (assuming approximately 15% drop-out) was sufficient to achieve approximately 90% overall power for demonstrating NI for the primary and secondary objectives. Sample size calculations assumed a one-sided significance level of 0.025 and were performed using nQuery Advisor^®^ 6.01.

DENV and YF GMTs and seroprotection/seropositivity rates are presented as means with corresponding 95% CIs. Titers below 10 were given an arbitrary value 5. For the primary immunogenicity endpoint, NI was concluded if the upper bound of the 95% CI of the difference in YF seroprotection rates (Group 1 minus Group 3) at Month 1 was <5%. The Newcombe score method was used to compute the 95% CI of the rate difference [36]. For the secondary objectives, NI of DENV GMTs when TAK-003 was concomitantly administered with YF-17D versus with placebo was concluded if the upper bound of the 95% CI for the GMT ratio (Group 2/Group 3) was <2. The NI of YF GMTs when YF-17D was concomitantly administered with TAK-003 versus with placebo was concluded if the upper bound of the 95% CI for the GMT ratio (Group 1/Group 3) was <2. For these assessments, ANOVA (analysis of variance) models were used, including the log-transformed value of titer as dependent variable and group as a factor. Assessment of GMTs and seroprotection/seropositivity rates of sequentially administered vaccines was performed with no formal statistical hypotheses tested.

Data are presented for the per-protocol sets (PPS). The YF PPS, i.e. all randomized participants who received planned Day 1 study vaccination(s) and for whom a valid pre-dose (baseline) and at least one post-dose measurement (Day 30) were available for YF immunogenicity assessments, and who had no major protocol violations, excluding those who were seroprotected for YF virus and/or who were seropositive for any DENV serotype at baseline, was used for assessment of the primary endpoint. The PPS, i.e. all randomized participants who received all planned study vaccinations, who provided a valid baseline measurement and at least one post-vaccination measurement for immunogenicity assessments, and had no major protocol violations, excluding those who were seroprotected for YF virus and/or who were seropositive for any DENV serotype at baseline, was used for assessment of all other immunogenicity endpoints. Safety was analyzed descriptively for the safety set, i.e. all participants who were randomized and received at least one dose of any study vaccines. All statistical analysis was performed using SAS version 9.4.

## Results

**Fig 2:**
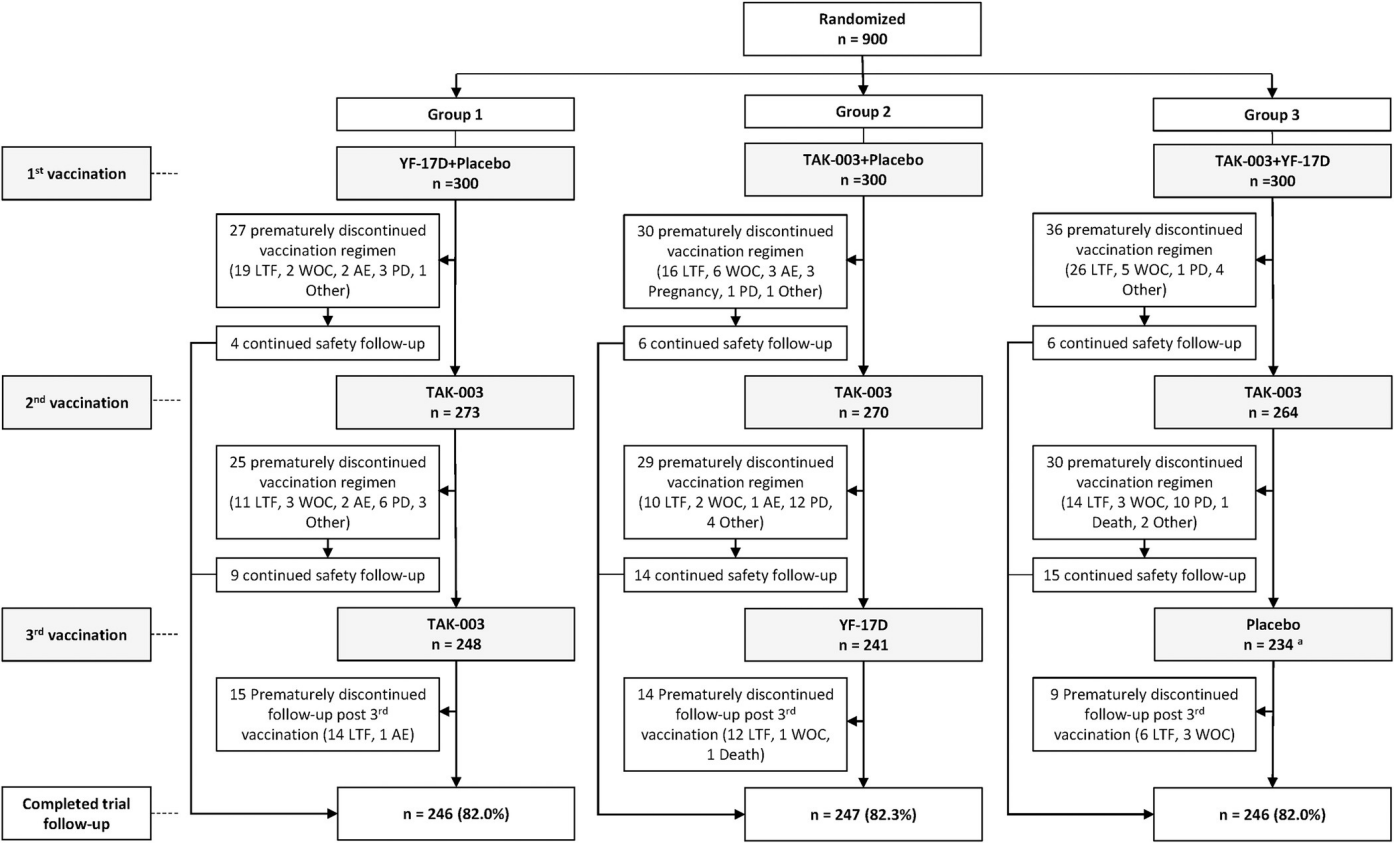
Participant flowchart. ^a^This includes 1 participant who received YF-17D vaccine instead of Placebo at 3^rd^ vaccination. AE, adverse event; LTF, lost to follow-up; PD, protocol deviation; TAK-003, tetravalent dengue vaccine candidate; WOC, withdrawal of consent; YF-17D, live attenuated yellow fever vaccine.

All 900 randomized participants received at least 1 vaccination. Of them, 723 (80.3%) received the 3 vaccinations, and 739 (82.1%) completed the study follow-up (**Fig 2**). Discontinuation rates were similar in each group, with majority of participants who did not complete the vaccination regimen and/or the study follow-up being lost to follow-up. Baseline characteristics were similar across the three treatment groups; the mean age was 41 years, 57% were female, and 66% were white (**Table 1**).

**Table 1:** Demographic and baseline characteristics (safety set).

	Group 1 YF-17D+P/ TAK-003/TAK-003 (N = 300)	Group 2 TAK-003+P/TAK-003/ YF-17D (N = 300)	Group 3 TAK-003+YF-17D/TAK-003/P (N = 299)^a^
Age (years)			
Mean (SD)	41.7 (12.4)	40.0 (12.9)	41.4 (12.7)
Gender (n[%])			
Male	133 (44.3)	130 (43.3)	124 (41.5)
Female	167 (55.7)	170 (56.7)	175 (58.5)
Ethnicity (n[%])			
Hispanic or Latino	26 (8.7)	25 (8.3)	30 (10.0)
Not Hispanic or Latino	272 (90.7)	270 (90.0)	266 (89.0)
Not reported/unknown	2 (0.7)	5 (1.6)	3 (1.0)
Race (n[%])			
Black or African American	77 (25.7)	103 (34.3)	84 (28.1)
White	205 (68.3)	186 (62.0)	200 (66.9)
Other	13 (4.3)	6 (2.0)	12 (4.0)
Not reported	5 (1.7)	5 (1.7)	3 (1.0)
Height (cm)			
Mean (SD)	171.3 (9.3)	170.1 (9.1)	170.5 (8.8)
Weight (kg)			
Mean (SD)	82.4 (15.4)	78.5 (14.6)	80.9 (16.9)
Body mass index (BMI) (kg/m^2^)			
Mean (SD)	28.0 (4.3)	27.1 (4.3)	27.7 (4.5)
Baseline seropositivity/seroprotection status			
Yellow fever seroprotection (n[%])^b^	34 (11.7)	21 (7.2)	23 (8.1)
DENV seropositive (n[%])^c^	34 (11.7)	20 (6.9)	28 (9.9)

DENV, dengue virus; P, placebo; SD, standard deviation; TAK-003, tetravalent dengue vaccine candidate; YF-17D, live attenuated yellow fever vaccine

^a^One participant was randomized to Group 3 but received a yellow fever vaccine instead of placebo at 3^rd^ vaccination so was excluded from the safety set

^b^Yellow fever seroprotection defined as reciprocal anti-yellow fever neutralizing antibody titer (plaque reduction neutralization test) ≥10

^c^DENV seropositivity defined as a reciprocal neutralizing antibody titer ≥10 for one or more DENV serotype at baseline (microneutralization assay)

### Immunogenicity–yellow fever

The primary study objective of NI of YF seroprotection rate at Month 1 for Groups 1 vs 3 was met (upper bound of the 95% CI for the between-group difference was <5%; **Table 2**). YF GMTs and seroprotection rates after YF-17D vaccination were high in all groups (**Table 3**). One month after the first vaccination (Month 1), seroprotection rates were 99.5% (95% CI: 97.4–100.0%) and 99.1 (96.9–99.9%) in Groups 1 (YF-17D plus placebo) and 3 (YF-17D plus TAK-003), respectively. Similarly, the seroprotection rate in Group 2 one month after the YF-17D vaccination (Month 7) was 98.4% (95.4–99.7%). In Group 2, seroprotection rates rose to 9.8% by Month 1 and 12.0% by Month 6, despite not having received the YF-17D vaccine yet.

**Table 2:** Non-inferiority comparisons (YF PPS and PPS).

	Group 1 YF-17D+P/TAK-003/TAK-003	Group 2 TAK-003+P/TAK-003/YF-17D	Group 3 TAK-003+YF-17D/TAK-003/P	Non-inferiority test
**Primary endpoint**				
Yellow fever seroprotection rate (SR), Month 1	*n = 211* 99.5 (97.4, 100.0)	–	*n = 229* 99.1 (96.9, 99.9)	Difference in SR: 0.40 (-1.85, **2.69**)
**Secondary endpoints**				
Yellow fever GMTs, Month 1	*n = 211* 4246 (3527, 5112)	–	*n = 229* 4322 (3653, 5114)	GMT ratio 1.00 (0.77, **1.26**)
DENV GMTs, Month 4		*n = 198*	*n = 177*	
DENV-1	–	297 (240, 368)	183 (146, 229)	GMT ratio: 1.6 (1.19, 2.22)
DENV-2	–	2616 (2133, 3208)	1948, (1640, 2313)	GMT ratio: 1.3 (1.03, **1.75**)
DENV-3	–	131 (111, 156)	105 (88, 124)	GMT ratio: 1.3 (0.99, **1.61**)
DENV-4	–	112 (95, 132)	98 (81, 117)	GMT ratio: 1.1 (0.89, **1.46**)

Bold indicates non-inferiority criteria met

DENV, dengue virus; GMT, geometric mean titer; P, placebo; PPS, per-protocol set; SR, seroprotection rate; TAK-003, tetravalent dengue vaccine candidate; YF-17D, live attenuated yellow fever vaccine

For the primary immunogenicity endpoint, non-inferiority (NI) was concluded if the upper bound of the 95% CI of the difference in YF seroprotection rates (Group 1 minus Group 3) at Month 1 was <5%.

For the secondary objectives, NI of YF GMTs when YF-17D was concomitantly administered with TAK-003 versus placebo was concluded if the upper bound of the 95% CI for the GMT ratio (Group 1/Group 3) was <2. NI of DENV GMTs when TAK-003 was concomitantly administered with YF-17D versus placebo was concluded if the upper bound of the 95% CI for the GMT ratio (Group 2/Group 3) was <2.

n represents the number of participants with data evaluated

**Table 3:** Geometric mean titers of anti-YF neutralizing antibodies and seroprotection rates (95% confidence interval) on Days 1 (Month 0), 30 (Month 1), 180 (Month 6), and 210 (Month 7) (YF PPS).

	Group 1 YF-17D+P/ TAK-003/TAK-003 (N = 223)	Group 2 TAK-003+P/TAK-003/ YF-17D (N = 255)	Group 3 TAK-003+YF-17D/TAK-003/P (N = 237)
**Geometric Mean titres** ^a^			
Month 0	*n = 223*	*n = 255*	*n = 237*
	5	5	5
Month 1	*n = 211*	*n = 234*	*n = 229*
	4246 (3527, 5112)	6.0 (5.5, 6.6)	4322 (3653, 5114)
Month 6	*n = 192*	*n = 208*	*n = 188*
	2712 (2229, 3301)	6.0 (5.5, 6.6)	1185 (974, 1440)
Month 7	*n = 171*	*n = 188*	*n = 176*
	2342 (1938, 2829)	3078 (2452, 3864)	1089 (888, 1336)
**Seroprotection Rates**			
Month 0	*n = 223*	*n = 233*	*n = 237*
	0	0	0
Month 1	*n = 211*	*n = 234*	*n = 229*
	99.5 (97.4, 100.0)	9.8 (6.3, 14.4)	99.1 (96.9, 99.9)
Month 6	*n = 192*	*n = 208*	*n = 188*
	99.5 (97.1, 100.0)	12.0 (7.9, 17.2)	98.9 (96.2, 99.9)
Month 7	*n = 171*	*n = 188*	*n = 176*
	100.0 (97.9, 100.0)	98.4 (95.4, 99.7)	98.9 (96.0, 99.9)

P, placebo; PPS, per-protocol set; TAK-003, tetravalent dengue vaccine candidate; YF-17D, live attenuated yellow fever vaccine

Yellow fever seroprotection defined as reciprocal anti-yellow fever neutralizing antibody titer (plaque reduction neutralization test) ≥10

n represents the number of participants with data evaluated

^a^Titers below 10 were imputed with an arbitrary value of 5

The secondary endpoint of NI of YF GMTs between Groups 1 and 3 at Month 1 was also met, as the upper bound of the 95% CI for the GMT ratio was <2 (**Table 2**). GMTs were of similar magnitude at Month 1 in Groups 1 and 3 (4246 versus 4322), although GMTs were lower in Group 3 than Group 1 by Months 6 and 7 (**Table 3**). YF GMT in Group 2 (sequential TAK-003 then YF-17D administration) was 3078 one month after YF-17D vaccination.

### Immunogenicity–dengue

**Fig 3:**
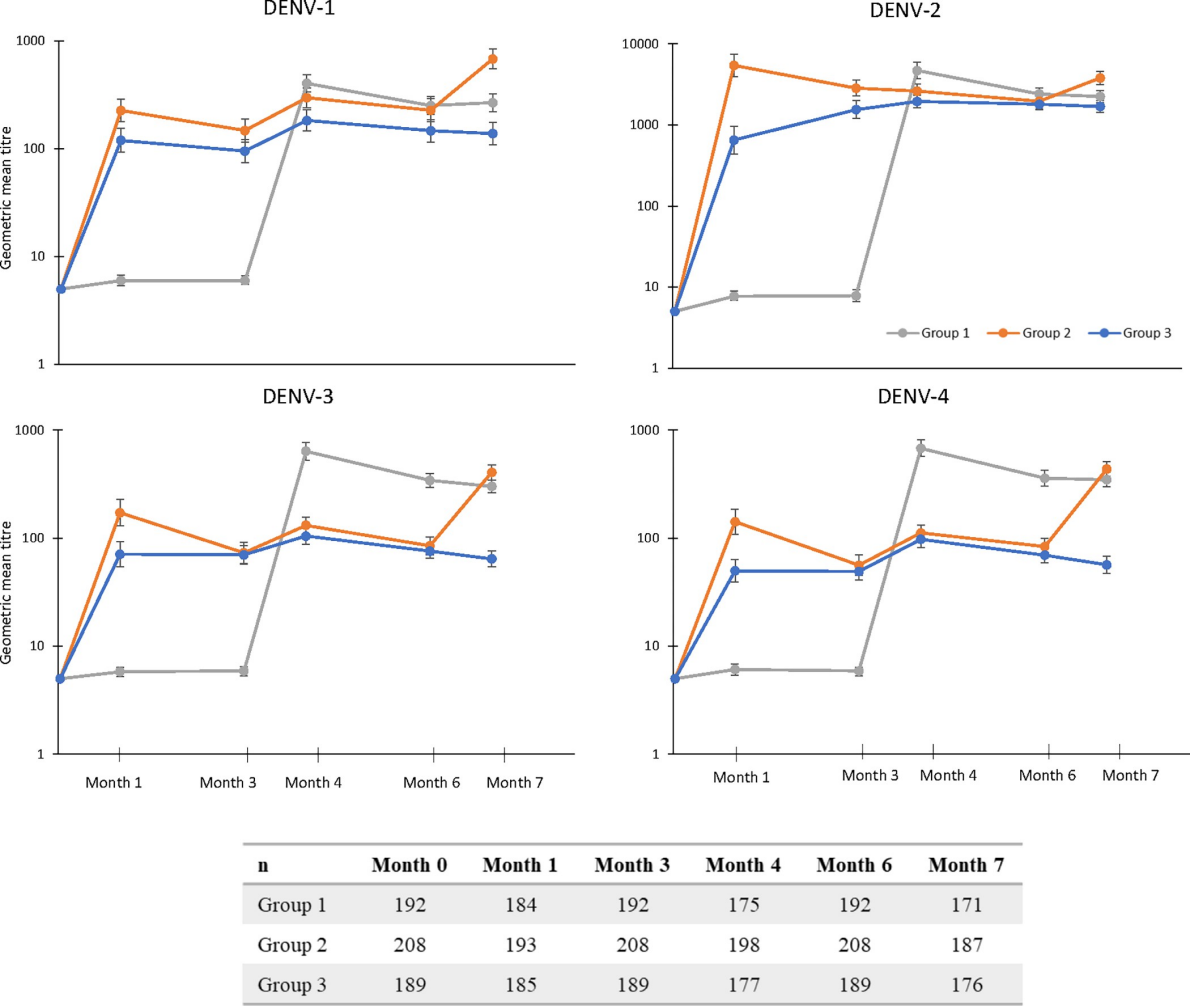
Geometric mean titers (GMTs) of neutralizing antibodies against each DENV serotype in each of the vaccine groups (PPS). Number of participants included at each time point are given in the table below the graphs. DENV, dengue virus; PPS, per-protocol set.

The secondary objective of NI of DENV neutralizing antibodies was concluded against DENV-2, -3, and -4, as the upper bound of the 95% CI for the GMT ratio between Groups 2 and 3 at Month 4 was <2 (**Table 2**). However, NI was not concluded against DENV-1, where higher GMTs were observed in Group 2 than Group 3. GMTs against all four DENV serotypes increased post-vaccination with TAK-003 in all three groups. Lower GMTs were observed in Group 3 (concomitant administration of YF-17D and TAK-003) than in Group 2 (sequential TAK-003 then YF-17D) against all serotypes, particularly between TAK-003 doses (**Fig 3 and S1 Table**). GMTs against DENV-3 and DENV-4 following the first and second dose of TAK-003 were of higher magnitude in Group 1 than those observed at either time point in the other two groups. In Group 2, GMTs against DENV-1, -3, and -4 were of higher magnitude post-YF-17D than just after the first or second dose of TAK-003. When sequentially administered, GMTs against DENV-1, -2, -3, and -4 one month after the second dose of TAK-003 were 268, 2248, 302, and 347 in Group 1 (Month 7); and 297, 2616, 131, and 121 in Group 2 (Month 4). GMTs in the concomitant administration group (Group 3) were 183, 1948, 105, and 98 (Month 4) (**S1 Table**).

**Fig 4:**
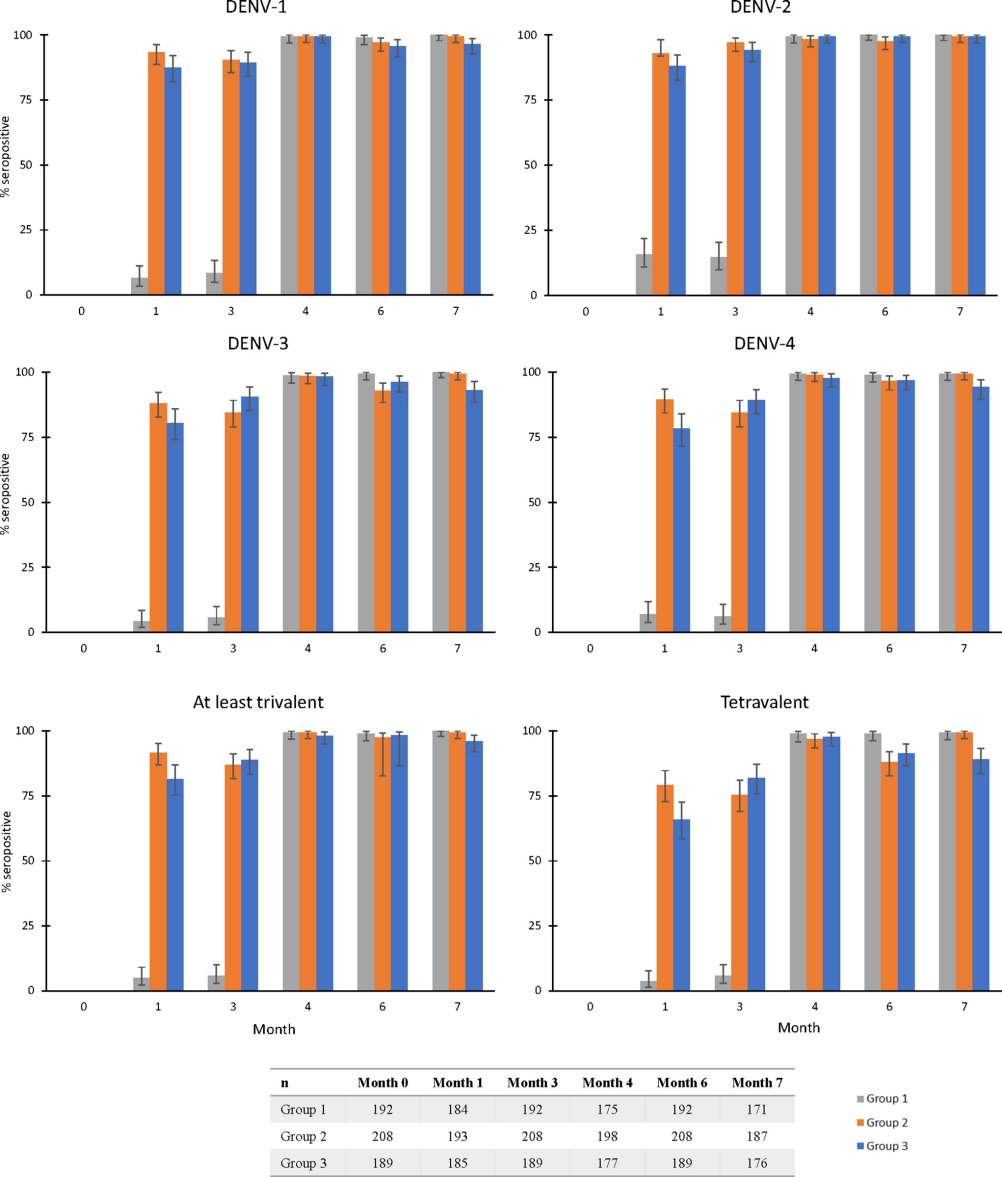
Seropositivity rates (95% confidence intervals) against individual and multiple DENV serotypes in each vaccine group (PPS). Number of participants included at each time point are given in the table below the graphs. DENV, dengue virus.

Seropositivity rates were high in all groups post-vaccination (**Fig 4 and S2 Table**). Tetravalent seropositivity rates by one month after the second dose of TAK-003 were 99.4% (96.8–100.0%) and 97.0% (93.5–98.9%), in the sequential administration Groups 1 and 2, and 97.7% (94.3–99.4%) in the concomitant administration Group 3. In Group 1, seropositivity rates increased to 4.3–15.8% across serotypes one month after receipt of the YF-17D vaccine but prior to receipt of TAK-003 (Month 1).

### Safety

Solicited local AEs were reported by 45.9%, 56.4%, and 60.3% of participants in Groups 1, 2, and 3, respectively (**Table 4**). The majority of these AEs were mild to moderate and did not persist beyond the diary period. The most frequently reported local AE across groups was injection-site pain, with rates varying depending on the vaccine received (TAK-003: 24.9%–42.2% of participants across vaccinations; YF-17D: 10.4–24.1%; placebo: 9.7–16.5%) (**S3–S5 Tables**). Rates of solicited systemic AEs were similar across groups (55.2%, 59.2%, and 60.6% in Groups 1, 2, and 3, respectively), and mostly mild to moderate (**Table 4**). The most frequent solicited systemic AE was headache. Rates of solicited AEs tended to decrease with subsequent vaccinations (**S6–S8 Tables**). Unsolicited AEs were reported by 20.3%, 25.3%, and 21.4% of participants in Groups 1, 2, and 3, respectively. The most commonly occurring AEs were injection site conditions and upper respiratory tract infections (**Table 5**), with rash reported after TAK-003 by four participants, and after YF-17D by one participant; all these events were considered mild to moderate.

**Table 4:** Number (%) of participants reporting solicited local adverse events (AEs) within 7 days or systemic AEs within 14 days after any vaccination overall and by severity (safety set).

	Group 1 YF-17D+P/ TAK-003/TAK-003 (N = 300)	Group 2 TAK-003+P/ TAK-003/YF-17D (N = 300)	Group 3 TAK-003+YF-17D/ TAK-003/P (N = 299) ^a^
**Solicited Local AEs, n**	290	289	282
Any	133 (45.9)	163 (56.4)	170 (60.3)
Severe	6 (2.1)	5 (1.7)	10 (3.5)
Pain	290	289	282
Any	123 (42.4)	147 (50.9)	153 (54.3)
Severe	4 (1.4)	5 (1.7)	8 (2.8)
Erythema	289	289	282
Any	51 (17.6)	80 (27.7)	76 (27.0)
Severe: >10 (cm)	2 (0.7)	0	2 (0.7)
Swelling	289	289	282
Any	18 (6.2)	28 (9.7)	28 (9.9)
Severe: >10 (cm)	1 (0.3)	0	1 (0.4)
**Solicited Systemic AEs, n**	290	289	282
Any^b^	160 (55.2)	171 (59.2)	171 (60.6)
Severe	28 (9.7)	30 (10.4)	21 (7.4)
Headache	290	289	282
Any	120 (41.4)	113 (39.1)	131 (46.5)
Severe	15 (5.2)	11 (3.8)	17 (6.0)
Asthenia	290	289	282
Any	78 (26.9)	77 (26.6)	74 (26.2)
Severe	10 (3.4)	9 (3.1)	9 (3.2)
Malaise	290	289	282
Any	95 (32.8)	83 (28.7)	87 (30.9)
Severe	12 (4.1)	18 (6.2)	10 (3.5)
Muscle pain (myalgia)	290	289	282
Any	98 (33.8)	106 (36.7)	106 (37.6)
Severe	10 (3.4)	12 (4.2)	10 (3.5)
Fever (body temperature in °C)	290	287	281
Any (≥38.0)	15 (5.2)	19 (6.6)	9 (3.2)
≥40.0	1 (0.3)	0	0

AE, adverse event; P, placebo; TAK-003, tetravalent dengue vaccine candidate; YF-17D, live attenuated yellow fever vaccine

n represents the number of participants with data evaluated

^a^One participant was randomized to Group 3 but received a yellow fever vaccine instead of placebo at 3^rd^ vaccination so was excluded from the safety set

^b^Fever is included in the “any” category but was not assessed by severity (mild/moderate/severe)

**Table 5:** Number (%) of participants reporting unsolicited adverse events (AEs) within 28 days post vaccination, and the number of events reported by MedDRA system organ class and preferred term. Data are shown for preferred terms in ≥2% of participants in any vaccine group (safety set).

	Group 1 YF-17D+P/ TAK-003/TAK-003 (N = 300)		Group 2 TAK-003+P/ TAK-003/YF-17D (N = 300)		Group 3 TAK-003+YF-17D/ TAK-003/P (N = 299)^a^	
**System Organ Class/Preferred Term**	**Events**	**Participants (%)**	**Events**	**Participants (%)**	**Events**	**Participants (%)**
**Any unsolicited AEs**	91	61 (20.3)	128	76 (25.3)	103	64 (21.4)
General disorders and administration site conditions	22	15 (5.0)	29	26 (8.7)	31	21 (7.0)
Injection site bruising	4	4 (1.3)	6	5 (1.7)	12	10 (3.3)
Injection site pruritus	5	3 (1.0)	7	7 (2.3)	10	8 (2.7)
Infections and infestations	27	23 (7.7)	31	30 (10.0)	27	26 (8.7)
Upper respiratory tract infection	5	5 (1.7)	12	12 (4.0)	4	4 (1.3)

AE, adverse event; P, placebo; TAK-003, tetravalent dengue vaccine candidate; YF-17D, live attenuated yellow fever vaccine; MedDRA, Medical Dictionary for Regulatory Activities

^a^One participant was randomized to Group 3 but received a yellow fever vaccine instead of placebo at 3^rd^ vaccination so was excluded from the safety set

MAAEs were reported by 14.3%, 15.3%, and 15.4% of participants in Groups 1, 2, and 3, respectively. Infections and infestations were the most commonly reported. Six participants reported MAAEs that were considered by the investigator as related to the study vaccinations (fatigue in Group 1; injection site pruritus and injection site bruising in Group 2; lymphangitis, arthralgia, back pain and night sweats in Group 3).

Across groups, twelve participants experienced AEs leading to withdrawal, including two deaths (cardiac arrest and diabetic ketoacidosis, and drug abuse), which were considered unrelated to the study vaccinations. SAEs were reported by 4.3%, 3.3%, and 2.3% of participants in Groups 1, 2, and 3, respectively (**S9 Table**). None of the SAEs were considered related to the study vaccinations.

## Discussion

In this study, NI of the immune response to YF-17D and TAK-003 was demonstrated for concurrent administration of the two vaccines compared to sequential vaccination, except against DENV-1. Some serological cross-reactivity was observed for both vaccines, prior to receipt of the second vaccine.

Marginally lower GMT against DENV-1 was observed one month after the second dose of TAK-003 (Month 4) in the group that received both vaccines (Group 3) compared with the group that received only TAK-003 (Group 2) at this time point. The clinical relevance of this observation is unknown as there is no correlate of protection established for dengue. However, the immune response against DENV-1 was similar to that observed in the clinical development program, specifically the pivotal efficacy trial in subjects who were DENV seronegative prior to vaccination [23]. The seroposivity rates for DENV-1 were also very similar at Month 4 across the 2 groups that had already received 2 doses of TAK-003 at this time point (Groups 2 and 3).

As both vaccines assessed in this study were live attenuated vaccines against flaviviruses, there was the potential for immunological interferences and development of cross-reactive antibodies, leading to altered immunogenicity of one or both of the vaccines. Cross-reactivity of flavivirus antibodies has been observed following sequential natural infections [28, 32, 37, 38], and in our study, YF seroprotection rates increased from zero to 10% after receipt of the first TAK-003 dose, before receipt of YF-17D, suggesting some degree of cross-reactivity. Additionally, co-administration of YF-17D and some other live attenuated vaccines, including other dengue vaccines, has been associated with reduced antibody response [29, 30, 34].

In our study, while NI of the response to the YF-17D vaccine, and to TAK-003 for DENV-2, -3, and -4 was demonstrated, DENV GMTs were numerically lower against all four serotypes 30 days after concomitant administration of the first dose of TAK-003 with YF-17D, compared with administration of TAK-003 alone. However, after the second dose of TAK-003, GMTs were mostly of similar magnitude in Groups 2 and 3, indicating that the effect was transient. The clinical implications of these observed interferences is unknown. Additionally, higher DENV GMTs following sequential vaccination (Groups 1 and 2), when compared with those following concomitant vaccination (Group 3), suggest a potential immune-enhancing effect of YF-17D (when administered prior to or after TAK-003) on TAK-003 antibody responses. Additionally, YF GMTs at Months 6 and 7 were higher in Group 1 than in Group 3 but were lower in Group 2 at Month 7 than in Groups 1 and 3 at Month 1, signaling that these immune interferences are complex, and may be influenced by the time and sequence of vaccination. As YF seroprotection rates remained near 100% at all time points post-sequential vaccination, this likely has no clinical impact for YF-17D vaccination. For TAK-003 vaccination, the clinical significance of this potential enhancing effect following sequential vaccination is currently unknown in absence of any immune correlates of protection for dengue.

The short duration of follow up for immunogenicity endpoints (one month following receipt of the last vaccination) was a limitation for this particular study. While antibody persistence and long-term safety has been reported from a four-year study in children and adolescents living in endemic areas of Latin America and Asia [21], the immunogenicity findings may be different in individuals living in non-endemic areas, who do not experience exposure to natural infection. Therefore, to assess long-term antibody persistence and safety in non-endemic areas, including the effect of a booster dose, an ongoing extension study is being performed in adolescents and adults who participated in two other phase 3 studies in the US and Mexico (NCT03999996). An additional limitation was the relatively high number of participants who prematurely discontinued the vaccination regimen and/or the trial follow-up, mainly as participants were lost to follow-up (Fig 2). The loss was balanced across the three treatment groups, which reduced the impact on the study findings, however the overall population size was slightly below that predicted for the power calculations.

One of the strengths of this study was that the design allowed analysis of concomitant and sequential administration of TAK-003 and YF-17D in a large group of participants who were seronegative for both diseases. As there is already a wealth of data available for both vaccines administered separately, we also chose not to include single vaccine groups to maximize the number of participants receiving the two vaccines concomitantly or sequentially. Performing the study in a non-endemic area for either virus allowed us to assess vaccine interactions without any additional impact of flavivirus exposure, either previously or during the study, as well as adding to the safety database of TAK-003 in seronegative vaccine recipients. Both vaccines in the study were well tolerated, and the safety profile of TAK-003 was similar to that observed in previous studies across baseline seronegative and seropositive participants [16–23]. Most solicited AEs were transient and mild to moderate in severity, with the most frequently reported AEs being injection site pain and headache, as observed previously in phase 2 studies [19–22]. Unsolicited AEs were mainly self-limiting and also similar to those observed in previous studies, and for other live-attenuated dengue vaccines [19–22,29].

In summary, no reduction in immune response to YF-17D vaccine, nor in immune responses to TAK-003 for DENV-2, -3, and -4, was observed when the 2 vaccines are co-administered. Marginally lower DENV-1 GMTs were observed. The clinical relevance of this is unknown but these titers remain similar to those observed in other trials, in particular in the pivotal efficacy trial that demonstrated the efficacy of TAK-003 against dengue cases (including those caused by DENV-1) in subjects who were DENV seronegative prior vaccination.

## Supporting information

S1 CONSORT checklist (DOC)

S1 Table Geometric mean titers (95% confidence interval) of neutralizing antibodies measured by microneutralization assay (MNT_50_) for each DENV serotype. (PDF)

S2 Table Seropositivity rates (95% confidence interval) against each DENV serotype. (PDF)

S3 Table Number (%) of participants reporting solicited local adverse events (AEs) within 7 days after the first vaccination by severity (safety set). (PDF)

S4 Table Number (%) of participants reporting solicited local adverse events (AEs) within 7 days after the second vaccination by severity (safety set). (PDF)

S5 Table Number (%) of participants reporting solicited local adverse events (AEs) within 7 days after the third vaccination by severity (safety set). (PDF)

S6 Table Number (%) of participants reporting solicited systemic adverse events (AEs) within 14 days after the first vaccination by severity (safety set). (PDF)

S7 Table Number (%) of participants reporting solicited systemic adverse events (AEs) within 14 days after the second vaccination by severity (safety set). (PDF)

S8 Table Number (%) of participants reporting solicited systemic adverse events (AEs) within 14 days after the third vaccination by severity (safety set). (PDF)

S9 Table Serious adverse events (SAEs) by MedDRA system organ class and preferred term after any vaccination (safety set). (PDF)
