# Supplementary material for: Immunogenicity and safety of concomitant and sequential administration of yellow fever YF-17D vaccine and tetravalent dengue vaccine candidate TAK-003: A phase 3 randomized, controlled study
Source: PLoS Negl Trop Dis. 2023 Mar 8;17(3):e0011124. doi: 10.1371/journal.pntd.0011124 (PMC9994689; doi:10.1371/journal.pntd.0011124)
Supplement: S1 Table — (PDF) [file pntd.0011124.s002.pdf]

Trial Visit	Serotype	Group 1<br>YF-17D+P/<br>TAK-003/TAK-003<br>(N=192)	Group 2<br>TAK-003+P/<br>TAK-003/YF-17D<br>(N=208)	Group 3<br>TAK-003+YF-17D/<br>TAK-003/P<br>(N=189)
Month 0, n		*192*	*208*	*189*
	DENV-1	5<br>(-,-)	5<br>(-,-)	5<br>(-,-)
	DENV-2	5<br>(-,-)	5<br>(-,-)	5<br>(-,-)
	DENV-3	5<br>(-,-)	5<br>(-,-)	5<br>(-,-)
	DENV-4	5<br>(-,-)	5<br>(-,-)	5<br>(-,-)
Month 1, n		*184*	*193*	*185*
	DENV-1	6<br>(5, 7)	226<br>(179, 286)	119<br>(93, 153)
	DENV-2	8<br>(7, 9)	5431<br>(3930, 7505)	651<br>(438, 967)
	DENV-3	6<br>(5, 6)	172<br>(129, 229)	71<br>(54, 93)
	DENV-4	6<br>(5, 7)	142<br>(109, 185)	50<br>(39, 63)
Month 3, n		*192*	*208*	*189*
	DENV-1	6<br>(6, 7)	148<br>(115, 190)	95<br>(74, 122)
	DENV-2	8<br>(7, 9)	2847<br>(2274, 3564)	1547<br>(1198, 1998)
	DENV-3	6<br>(5, 7)	73<br>(58, 92)	70<br>(57, 86)
	DENV-4	6<br>(5, 6)	56<br>(45, 70)	49<br>(41, 59)
Month 4, n		*175*	*198*	*177*
	DENV-1	404<br>(335, 487)	297<br>(240, 368)	183<br>(146, 229)
	DENV-2	4691<br>(3689, 5966)	2616<br>(2133, 3208)	1948<br>(1640, 2313)
	DENV-3	636<br>(526, 771)	131<br>(111, 156)	105<br>(88, 124)
	DENV-4	679<br>(569, 809)	112<br>(95, 132)	98<br>(81, 117)
Month 6, n		*192*	*208*	*189*
	DENV-1	251<br>(209, 302)	227<br>(177, 290)	147<br>(116, 187)
	DENV-2	2414<br>(2039, 2857)	1959<br>(1584, 2424)	1800<br>(1536, 2110)
	DENV-3	342<br>(295, 398)	85<br>(70, 103)	76<br>(65, 88)
	DENV-4	358<br>(303, 424)	84<br>(70, 100)	70<br>(59, 82)
Month 7, n		*171*	*187*	*176*
	DENV-1	268<br>(222, 323)	680<br>(550, 841)	138<br>(108, 176)
	DENV-2	2248<br>(1902, 2657)	3798<br>(3139, 4595)	1688<br>(1432, 1990)
	DENV-3	302<br>(263, 347)	406<br>(345, 478)	64<br>(54, 76)
	DENV-4	347<br>(298, 406)	437<br>(375, 509)	57<br>(47, 68)

P, placebo; TAK-003, tetravalent dengue vaccine candidate; YF-17D, live attenuated yellow fever vaccine
Titers below 10 were imputed with an arbitrary value of 5
