# Supplementary material for: Immunogenicity and safety of concomitant and sequential administration of yellow fever YF-17D vaccine and tetravalent dengue vaccine candidate TAK-003: A phase 3 randomized, controlled study
Source: PLoS Negl Trop Dis. 2023 Mar 8;17(3):e0011124. doi: 10.1371/journal.pntd.0011124 (PMC9994689; doi:10.1371/journal.pntd.0011124)
Supplement: S2 Table — (PDF) [file pntd.0011124.s003.pdf]

Trial Visit	Serotype	Group 1<br>YF-17D+P/<br>TAK-003/TAK-003<br>(N=192)	Group 2<br>TAK-003+P/<br>TAK-003/YF-17D<br>(N=208)	Group 3<br>TAK-003+YF-17D/<br>TAK-003/P<br>(N=189)
Month 0, n		*192*	*208*	*189*
	DENV-1	0<br>(-,-)	0<br>(-,-)	0<br>(-,-)
	DENV-2	0<br>(-,-)	0<br>(-,-)	0<br>(-,-)
	DENV-3	0<br>(-,-)	0<br>(-,-)	0<br>(-,-)
	DENV-4	0<br>(-,-)	0<br>(-,-)	0<br>(-,-)
	At least trivalent	0<br>(-,-)	0<br>(-,-)	0<br>(-,-)
	Tetravalent	0<br>(-,-)	0<br>(-,-)	0<br>(-,-)
Month 1, n		*184*	*193*	*185*
	DENV-1	6.5<br>(3.4, 11.1)	93.3<br>(88.8, 96.4)	87.6<br>(81.9, 92.0)
	DENV-2	15.8<br>(10.8, 21.8)	95.9<br>(92.0, 98.2)	88.1<br>(82.6, 92.4)
	DENV-3	4.3<br>(1.9, 8.4)	88.1<br>(82.7, 92.3)	80.5<br>(74.1, 86.0)
	DENV-4	7.1<br>(3.8, 11.8)	89.6<br>(84.4, 93.6)	78.4<br>(71.7, 84.1)
	At least trivalent	4.9<br>(2.3, 9.1)	91.7<br>(86.9, 95.2)	81.6<br>(75.3, 86.9)
	Tetravalent	3.8<br>(1.5, 7.7)	79.3<br>(72.9, 84.8)	65.9<br>(58.6, 72.7)
Month 3, n		*192*	*208*	*189*
	DENV-1	8.3<br>(4.8, 13.2)	90.4<br>(85.5, 94.0)	89.4<br>(84.1, 93.4)
	DENV-2	14.6<br>(9.9, 20.4)	97.1<br>(93.8, 98.9)	94.2<br>(89.8, 97.1)
	DENV-3	5.7<br>(2.9, 10.0)	84.6<br>(79.0, 89.2)	90.5<br>(85.4, 94.3)
	DENV-4	6.3<br>(3.3, 10.7)	84.6<br>(79.0, 89.2)	89.4<br>(84.1, 93.4)
	At least trivalent	5.7<br>(2.9, 10.0)	87.0<br>(81.7, 91.3)	88.9<br>(83.5, 93.0)
	Tetravalent	5.7<br>(2.9, 10.0)	75.5<br>(69.1, 81.2)	82.0<br>(75.8, 87.2)
Month 4, n		*175*	*198*	*177*
	DENV-1	99.4<br>(96.9, 100.0)	99.5<br>(97.2, 100.0)	99.4<br>(96.9, 100.0)
	DENV-2	99.4<br>(96.9, 100.0)	98.5<br>(95.6, 99.7)	99.4<br>(96.9, 100.0)
	DENV-3	98.9<br>(95.9, 99.9)	98.5<br>(95.6, 99.7)	98.3<br>(95.1, 99.6)
	DENV-4	99.4<br>(96.9, 100.0)	99.0<br>(96.4, 99.9)	97.7<br>(94.3, 99.4)
	At least trivalent	99.4<br>(96.9, 100.0)	99.5<br>(97.2, 100.0)	98.3<br>(95.1, 99.6)
	Tetravalent	98.9<br>(95.9, 99.9)	97.0<br>(93.5, 98.9)	97.7<br>(94.3, 99.4)

(continued)

Month 6, n		*192*	*208*	*189*
	DENV-1	99.0<br>(96.3, 99.9)	97.1<br>(93.8, 98.9)	95.8<br>(91.8, 98.2)
	DENV-2	100.0<br>(98.1, 100.0)	97.6<br>(94.5, 99.2)	99.5<br>(97.1, 100.0)
	DENV-3	99.5<br>(97.1, 100.0)	92.8<br>(88.4, 95.9)	96.3<br>(92.5, 98.5)
	DENV-4	99.0<br>(96.3, 99.9)	96.6<br>(93.2, 98.6)	96.8<br>(93.2, 98.8)
	At least trivalent	99.0<br>(96.3, 99.9)	97.6<br>(94.5, 99.2)	98.4<br>(95.4, 99.7)
	Tetravalent	99.0<br>(96.3, 99.9)	88.0<br>(82.8, 92.1)	91.5<br>(86.6, 95.1)
Month 7, n		*171*	*187*	*176*
	DENV-1	100.0<br>(97.9, 100.0)	99.5<br>(97.1, 100.0)	96.6<br>(92.7, 98.7)
	DENV-2	100.0<br>(97.9, 100.0)	99.5<br>(97.1, 100.0)	99.4<br>(96.9, 100.0)
	DENV-3	100.0<br>(97.9, 100.0)	99.5<br>(97.1, 100.0)	93.2<br>(88.4, 96.4)
	DENV-4	99.4<br>(96.8, 100.0)	99.5<br>(97.1, 100.0)	94.3<br>(89.8, 97.2)
	At least trivalent	100.0<br>(97.9, 100.0)	99.5<br>(97.1, 100.0)	96.0<br>(92.0, 98.4)
	Tetravalent	99.4<br>(96.8, 100.0)	99.5<br>(97.1, 100.0)	89.2<br>(83.7, 93.4)

P, placebo; TAK-003, tetravalent dengue vaccine candidate; YF-17D, live attenuated yellow fever vaccine
