# Supplementary material for: Immunogenicity and safety of concomitant and sequential administration of yellow fever YF-17D vaccine and tetravalent dengue vaccine candidate TAK-003: A phase 3 randomized, controlled study
Source: PLoS Negl Trop Dis. 2023 Mar 8;17(3):e0011124. doi: 10.1371/journal.pntd.0011124 (PMC9994689; doi:10.1371/journal.pntd.0011124)
Supplement: S3 Table — (PDF) [file pntd.0011124.s004.pdf]

	Group 1<br>YF-17D+P/<br>TAK-003/TAK-003<br>(N=300)		Group 2<br>TAK-003+P/<br>TAK-003/YF-17D<br>(N=300)		Group 3<br>TAK-003+YF-17D/<br>TAK-003/P<br>(N=300)	
**First Vaccination**	**YF-17D**	**Placebo**	**Placebo**	**TAK-003**	**YF-17D**	**TAK-003**
Solicited Local AEs, n	289	289	285	285	282	282
Any	38 (13.1)	38 (13.1)	48 (16.8)	126 (44.2)	77 (27.3)	138 (48.9)
Severe	1 (0.3)	1 (0.3)	1 (0.4)	1 (0.4)	2 (0.7)	5 (1.8)
Pain, n	289	289	285	285	282	282
Any	35 (12.1)	37 (12.8)	47 (16.5)	114 (40.0)	68 (24.1)	119 (42.2)
Severe	0	1 (0.3)	1 (0.4)	1 (0.4)	2 (0.7)	4 (1.4)
Erythema, n	288	288	284	285	282	282
Any	7 (2.4)	2 (0.7)	5 (1.8)	56 (19.6)	11 (3.9)	55 (19.5)
Severe: >10 (cm)	1 (0.3)	0	0	0	0	1 (0.4)
Swelling, n	287	287	285	285	282	282
Any	2 (0.7)	1 (0.3)	3 (1.1)	12 (4.2)	6 (2.1)	15 (5.3)
Severe: >10 (cm)	1 (0.3)	0	0	0	0	1 (0.4)

P, placebo; TAK-003, tetravalent dengue vaccine candidate; YF-17D, live attenuated yellow fever vaccine
