# Supplementary material for: Immunogenicity and safety of concomitant and sequential administration of yellow fever YF-17D vaccine and tetravalent dengue vaccine candidate TAK-003: A phase 3 randomized, controlled study
Source: PLoS Negl Trop Dis. 2023 Mar 8;17(3):e0011124. doi: 10.1371/journal.pntd.0011124 (PMC9994689; doi:10.1371/journal.pntd.0011124)
Supplement: S4 Table — (PDF) [file pntd.0011124.s005.pdf]

	Group 1<br>YF-17D+P/<br>TAK-003/TAK-003<br>(N=273)	Group 2<br>TAK-003+P/<br>TAK-003/YF-17D<br>(N=270)	Group 3<br>TAK-003+YF-17D/<br>TAK-003/P<br>(N=264)
**Second Vaccination**	**TAK-003**	**TAK-003**	**TAK-003**
Solicited Local AEs, n	264	256	252
Any	82 (31.1)	92 (35.9)	90 (35.7)
Severe	3 (1.1)	2 (0.8)	3 (1.2)
Pain, n	263	256	252
Any	73 (27.8)	77 (30.1)	80 (31.7)
Severe	2 (0.8)	2 (0.8)	3 (1.2)
Erythema, n	261	256	252
Any	32 (12.3)	37 (14.5)	33 (13.1)
Severe: >10 (cm)	1 (0.4)	0	0
Swelling, n	262	256	252
Any	7 (2.7)	15 (5.9)	14 (5.6)
Severe: >10 (cm)	0	0	0

P, placebo; TAK-003, tetravalent dengue vaccine candidate; YF-17D, live attenuated yellow fever vaccine
