# Supplementary material for: Immunogenicity and safety of concomitant and sequential administration of yellow fever YF-17D vaccine and tetravalent dengue vaccine candidate TAK-003: A phase 3 randomized, controlled study
Source: PLoS Negl Trop Dis. 2023 Mar 8;17(3):e0011124. doi: 10.1371/journal.pntd.0011124 (PMC9994689; doi:10.1371/journal.pntd.0011124)
Supplement: S5 Table — (PDF) [file pntd.0011124.s006.pdf]

| | Group 1 YF-17D+P/ TAK-003/TAK-003 (N=248) | Group 2 TAK-003+P/ TAK-003/YF-17D (N=241) | Group 3 TAK-003+YF-17D/ TAK-003/P (N=233) |
|---|---|---|---|
| **Third Vaccination** | **TAK-003** | **YF-17D** | **P** |
| Solicited Local AEs | 237 | 230 | 227 |
| Any | 66 (27.8) | 24 (10.4) | 24 (10.6) |
| Severe | 1 (0.4) | 1 (0.4) | 3 (1.3) |
| Pain, n | 237 | 230 | 227 |
| Any | 59 (24.9) | 24 (10.4) | 22 (9.7) |
| Severe | 1 (0.4) | 1 (0.4) | 2 (0.9) |
| Erythema, n | 236 | 229 | 226 |
| Any | 23 (9.7) | 1 (0.4) | 2 (0.9) |
| Severe: >10 (cm) | 0 | 0 | 1 (0.4) |
| Swelling, n | 236 | 230 | 227 |
| Any | 10 (4.2) | 0 | 0 |
| Severe: >10 (cm) | 0 | 0 | 0 |

P, placebo; TAK-003, tetravalent dengue vaccine candidate; YF-17D, live attenuated yellow fever vaccine

Note: one participant in Group 3 received a yellow fever vaccination instead of placebo at 3rd vaccination and is excluded from Group 3 in the safety set for 'after third vaccination'
