# Supplementary material for: Immunogenicity and safety of concomitant and sequential administration of yellow fever YF-17D vaccine and tetravalent dengue vaccine candidate TAK-003: A phase 3 randomized, controlled study
Source: PLoS Negl Trop Dis. 2023 Mar 8;17(3):e0011124. doi: 10.1371/journal.pntd.0011124 (PMC9994689; doi:10.1371/journal.pntd.0011124)
Supplement: S6 Table — (PDF) [file pntd.0011124.s007.pdf]

	Group 1<br>YF-17D+P/<br>TAK-003/TAK-003<br>(N=300)	Group 2<br>TAK-003+P/<br>TAK-003/YF-17D<br>(N=300)	Group 3<br>TAK-003+YF-17D/<br>TAK-003/P<br>(N=299)
**First Vaccination**	**YF-17D+P**	**TAK-003+P**	**TAK-003+YF-17D**
Solicited Systemic AEs, n	289	285	282
Any[a]	126 (43.6)	146 (51.2)	147 (52.1)
Severe	11 (3.8)	20 (7.0)	16 (5.7)
Headache, n	289	285	281
Any	92 (31.8)	94 (33.0)	112 (39.9)
Severe	5 (1.7)	6 (2.1)	13 (4.6)
Asthenia, n	289	285	281
Any	51 (17.6)	60 (21.1)	57 (20.3)
Severe	6 (2.1)	6 (2.1)	5 (1.8)
Malaise, n	289	285	282
Any	60 (20.8)	59 (20.7)	69 (24.5)
Severe	5 (1.7)	13 (4.6)	6 (2.1)
Muscle Pain (Myalgia), n	289	285	282
Any	67 (23.2)	84 (29.5)	83 (29.4)
Severe	6 (2.1)	8 (2.8)	5 (1.8)
Fever, n	288	281	279
Any (≥38.0)	4 (1.4)	17 (6.0)	3 (1.1)
≥40.0°C	1 (0.3)	0	0

P, placebo; TAK-003, tetravalent dengue vaccine candidate; YF-17D, live attenuated yellow fever vaccine
[a]Fever is included in the "any" category but was not assessed by severity (mild/moderate/severe)
