# Supplementary material for: Immunogenicity and safety of concomitant and sequential administration of yellow fever YF-17D vaccine and tetravalent dengue vaccine candidate TAK-003: A phase 3 randomized, controlled study
Source: PLoS Negl Trop Dis. 2023 Mar 8;17(3):e0011124. doi: 10.1371/journal.pntd.0011124 (PMC9994689; doi:10.1371/journal.pntd.0011124)
Supplement: S7 Table — (PDF) [file pntd.0011124.s008.pdf]

	Group 1 YF-17D+P/ TAK-003/TAK-003 (N=273)	Group 2 TAK-003+P/ TAK-003/YF-17D (N=270)	Group 3 TAK-003+YF-17D/ TAK-003/P (N=264)
**Second Vaccination**	**TAK-003**	**TAK-003**	**TAK-003**
Solicited Systemic AEs, n	264	256	252
Any[a]	90 (34.1)	77 (30.1)	80 (31.7)
Severe	13 (4.9)	8 (3.1)	11 (4.4)
Headache, n	264	256	252
Any	60 (22.7)	40 (15.6)	53 (21.0)
Severe	8 (3.0)	3 (1.2)	5 (2.0)
Asthenia, n	264	256	252
Any	35 (13.3)	22 (8.6)	29 (11.5)
Severe	3 (1.1)	4 (1.6)	6 (2.4)
Malaise, n	264	256	252
Any	37 (14.0)	25 (9.8)	25 (9.9)
Severe	5 (1.9)	3 (1.2)	4 (1.6)
Muscle Pain (Myalgia), n	264	256	252
Any	55 (20.8)	48 (18.8)	51 (20.2)
Severe	3 (1.1)	3 (1.2)	7 (2.8)
Fever, n	260	250	248
Any (≥38.0)	8 (3.1)	3 (1.2)	3 (1.2)
≥40.0 °C	0	0	0

P, placebo; TAK-003, tetravalent dengue vaccine candidate; YF-17D, live attenuated yellow fever vaccine
[a]Fever is included in the “any” category but was not assessed by severity (mild/moderate/severe)
