# Supplementary material for: Immunogenicity and safety of concomitant and sequential administration of yellow fever YF-17D vaccine and tetravalent dengue vaccine candidate TAK-003: A phase 3 randomized, controlled study
Source: PLoS Negl Trop Dis. 2023 Mar 8;17(3):e0011124. doi: 10.1371/journal.pntd.0011124 (PMC9994689; doi:10.1371/journal.pntd.0011124)
Supplement: S8 Table — (PDF) [file pntd.0011124.s009.pdf]

	Group 1<br>YF-17D+P/<br>TAK-003/TAK-003<br>(N=248)	Group 2<br>TAK-003+P/<br>TAK-003/YF-17D<br>(N=241)	Group 3<br>TAK-003+YF-17D/<br>TAK-003/P<br>(N=233)
**Third Vaccination**	**TAK-003**	**YF-17D**	**P**
Solicited Systemic AEs, n	238	230	228
Any[a]	60 (25.2)	63 (27.4)	53 (23.2)
Severe	6 (2.5)	4 (1.7)	5 (2.2)
Headache, n	238	230	228
Any	41 (17.2)	40 (17.4)	46 (20.2)
Severe	2 (0.8)	3 (1.3)	4 (1.8)
Asthenia, n	238	230	228
Any	25 (10.5)	21 (9.1)	16 (7.0)
Severe	1 (0.4)	0	1 (0.4)
Malaise, n	238	230	228
Any	27 (11.3)	31 (13.5)	26 (11.4)
Severe	3 (1.3)	2 (0.9)	2 (0.9)
Muscle pain (myalgia), n	238	230	228
Any	30 (12.6)	28 (12.2)	18 (7.9)
Severe	3 (1.3)	1 (0.4)	1 (0.4)
Fever, n	234	228	227
Any (≥38.0)	3 (1.3)	0	3 (1.3)
≥40.0 °C	0	0	0

P, placebo; TAK-003, tetravalent dengue vaccine candidate; YF-17D, live attenuated yellow fever vaccine

Note: one participant in Group 3 received a yellow fever vaccination instead of placebo at 3rd vaccination and is excluded from Group 3 in the safety set for 'after third vaccination'

[a]Fever is included in the “any” category but was not assessed by severity (mild/moderate/severe)
