# Supplementary material for: Immunogenicity and safety of concomitant and sequential administration of yellow fever YF-17D vaccine and tetravalent dengue vaccine candidate TAK-003: A phase 3 randomized, controlled study
Source: PLoS Negl Trop Dis. 2023 Mar 8;17(3):e0011124. doi: 10.1371/journal.pntd.0011124 (PMC9994689; doi:10.1371/journal.pntd.0011124)
Supplement: S9 Table — (PDF) [file pntd.0011124.s010.pdf]

System Organ Class/Preferred Term	Group 1 YF-17D+P/ TAK-003/TAK-003 (N=300)		Group 2 TAK-003+P/ TAK-003/YF-17D (N=300)		Group 3 TAK-003+YF-17D/ TAK-003/P (N=299)	
	Events	Participants (%)	Events	Participants (%)	Events	Participants (%)
Any Serious Adverse Events	14	13 (4.3)	15	10 (3.3)	9	7 (2.3)
Cardiac disorders	0	0	1	1 (0.3)	0	0
Cardiac arrest	0	0	1	1 (0.3)	0	0
Endocrine disorders	0	0	0	0	1	1 (0.3)
Hypothyroidism	0	0	0	0	1	1 (0.3)
Gastrointestinal disorders	3	3 (1.0)	4	3 (1.0)	0	0
Abdominal pain	0	0	1	1 (0.3)	0	0
Alcoholic pancreatitis	0	0	1	1 (0.3)	0	0
Inguinal hernia	1	1 (0.3)	0	0	0	0
Large intestinal obstruction	0	0	1	1 (0.3)	0	0
Megacolon	0	0	1	1 (0.3)	0	0
Peptic ulcer	1	1 (0.3)	0	0	0	0
Peptic ulcer hemorrhage	1	1 (0.3)	0	0	0	0
General disorders and administration site conditions	0	0	1	1 (0.3)	0	0
Systemic inflammatory response syndrome	0	0	1	1 (0.3)	0	0
Hepatobiliary disorders	0	0	0	0	1	1 (0.3)
Cholecystitis acute	0	0	0	0	1	1 (0.3)
Immune system disorders	0	0	1	1 (0.3)	0	0
Allergy to arthropod sting	0	0	1	1 (0.3)	0	0
Infections and infestations	1	1 (0.3)	2	2 (0.7)	2	2 (0.7)
Pneumonia	0	0	1	1 (0.3)	2	2 (0.7)
Abscess intestinal	0	0	1	1 (0.3)	0	0
Urinary tract infection	1	1 (0.3)	0	0	0	0
Injury, poisoning and procedural complications	1	1 (0.3)	2	2 (0.7)	1	1 (0.3)
Ankle fracture	0	0	1	1 (0.3)	0	0
Gastrointestinal anastomotic leak	0	0	1	1 (0.3)	0	0
Humerus fracture	1	1 (0.3)	0	0	0	0
Traumatic fracture	0	0	0	0	1	1 (0.3)
Metabolism and nutrition disorders	0	0	1	1 (0.3)	0	0
Diabetic ketoacidosis	0	0	1	1 (0.3)	0	0
Musculoskeletal and connective tissue disorders	1	1 (0.3)	0	0	0	0
Musculoskeletal chest pain	1	1 (0.3)	0	0	0	0

(continued)

Neoplasms benign, malignant and unspecified (incl cysts and polyps)	6	6 (2.0)	0	0	0	0
Intraductal proliferative breast lesion	2	2 (0.7)	0	0	0	0
Aesthesioneuroblastoma	1	1 (0.3)	0	0	0	0
Invasive ductal breast carcinoma	1	1 (0.3)	0	0	0	0
Neuroendocrine tumor	1	1 (0.3)	0	0	0	0
Papillary thyroid cancer	1	1 (0.3)	0	0	0	0
Pregnancy, puerperium and perinatal conditions	0	0	1	1 (0.3)	1	1 (0.3)
Abortion spontaneous	0	0	1	1 (0.3)	1	1 (0.3)
Psychiatric disorders	1	1 (0.3)	1	1 (0.3)	2	2 (0.7)
Anxiety	1	1 (0.3)	1	1 (0.3)	0	0
Alcohol withdrawal syndrome	0	0	0	0	1	1 (0.3)
Drug abuse	0	0	0	0	1	1 (0.3)
Respiratory, thoracic and mediastinal disorders	0	0	1	1 (0.3)	1	1 (0.3)
Pulmonary embolism	0	0	1	1 (0.3)	1	1 (0.3)
Social circumstances	1	1 (0.3)	0	0	0	0
Organ donor	1	1 (0.3)	0	0	0	0

P, placebo; TAK-003, tetravalent dengue vaccine candidate; YF-17D, live attenuated yellow fever vaccine; MedDRA, Medical Dictionary for Regulatory Activities

Note: one participant in Group 3 received a yellow fever vaccination instead of placebo at 3rd vaccination and is excluded from Group 3 in the safety set for 'after any vaccination'
